# Machine Learning and Deep Learning Applications in Multiple Myeloma Diagnosis, Prognosis, and Treatment Selection

**DOI:** 10.3390/cancers14030606

**Published:** 2022-01-25

**Authors:** Alessandro Allegra, Alessandro Tonacci, Raffaele Sciaccotta, Sara Genovese, Caterina Musolino, Giovanni Pioggia, Sebastiano Gangemi

**Affiliations:** 1 Division of Hematology, Department of Human Pathology in Adulthood and Childhood “Gaetano Barresi”, University of Messina, 98125 Messina, Italy; sciaccottaraffaele@gmail.com (R.S.); cmusolino@unime.it (C.M.); 2 Clinical Physiology Institute, National Research Council of Italy (IFC-CNR), 56124 Pisa, Italy; atonacci@ifc.cnr.it; 3 Institute for Biomedical Research and Innovation (IRIB), National Research Council of Italy (CNR), 98164 Messina, Italy; sara.genovese@cnr.it (S.G.); giovanni.pioggia@cnr.it (G.P.); 4 Department of Clinical and Experimental Medicine, Unit and School of Allergy and Clinical Immunology, University of Messina, 98125 Messina, Italy; gangemis@unime.it

**Keywords:** artificial intelligence, machine learning, deep learning, multiple myeloma, prognosis, diagnosis, chemotherapy, bone disease

## Abstract

**Simple Summary:**

Multiple myeloma is a malignant neoplasm of plasma cells with complex pathogenesis. With major progresses in multiple myeloma research, it is essential that we reconsider our methods for diagnosing and monitoring multiple myeloma disease. This fact needs the integration of serology, histology, radiology, and genetic data; therefore, multiple myeloma study has generated massive quantities of granular high-dimensional data exceeding human understanding. With improved computational techniques, artificial intelligence tools for data processing and analysis are becoming more and more relevant. Artificial intelligence represents a wide set of algorithms for which machine learning and deep learning are presently among the most impactful. This review focuses on artificial intelligence applications in multiple myeloma research, first illustrating machine learning and deep learning procedures and workflow, followed by how these algorithms are used for multiple myeloma diagnosis, prognosis, bone lesions identification, and evaluation of response to the treatment.

**Abstract:**

Artificial intelligence has recently modified the panorama of oncology investigation thanks to the use of machine learning algorithms and deep learning strategies. Machine learning is a branch of artificial intelligence that involves algorithms that analyse information, learn from that information, and then employ their discoveries to make abreast choice, while deep learning is a field of machine learning basically represented by algorithms inspired by the organization and function of the brain, named artificial neural networks. In this review, we examine the possibility of the artificial intelligence applications in multiple myeloma evaluation, and we report the most significant experimentations with respect to the machine and deep learning procedures in the relevant field. Multiple myeloma is one of the most common haematological malignancies in the world, and among them, it is one of the most difficult ones to cure due to the high occurrence of relapse and chemoresistance. Machine learning- and deep learning-based studies are expected to be among the future strategies to challenge this negative-prognosis tumour via the detection of new markers for their prompt discovery and therapy selection and by a better evaluation of its relapse and survival.

## 1. Introduction

### General Considerations on Machine Learning and Deep Learning

Artificial intelligence (AI) includes several tools and algorithms with the specific ambition to computationally imitate the human intelligence. AI might use various algorithms derived from the subfields of machine learning (ML) or deep learning (DL) to push forward the computerisation of human experts’ tasks, able to lead to a substantial and concrete effect in healthcare. Recently, the medical applications of AI have extended not only to the clinical research but also to translational medicine and clinical procedures of different diseases, including tumours [1,2,3,4].

Overall, ML aims at generating informed evaluations by detecting relationships in information employing numerical algorithms, with these procedures presenting the advantage of being able to computerise the method of hypothesis construction. ML algorithms have integrated and, in some instances, modified the conventional statistical methodologies [5].

DL is a subfield of ML enthused by the configuration of the brain relying on the so-called artificial neural networks. Basically, it structures algorithms into layers in order to create artificial neural networks (ANN), able to learn and take intelligent decision on their own. In fact, differently from ML, in the case of DL, the algorithm will be able to determine the accuracy of a given prediction based on their own ANN, not requiring any sort of human intervention as occurring; instead, with ML and in this, DL resembles more the functioning of a real “brain,” making from it a sort of “human-like” AI method.

The evaluation of DL procedures’ results has demonstrated that, despite the general higher computational load they require, in many cases, this sort of practices overtakes other, more traditional ML methods [6]. DL structures have also been generated and employed for tumour detection, and they have been used to face the challenge of prefiguring the drug effects in several types of tumours [7,8,9].

**Figure 1:**
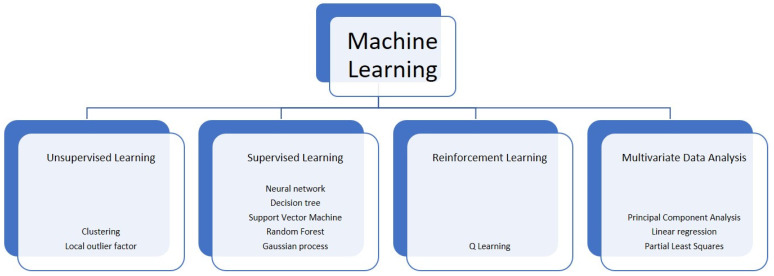
Some of the main computational techniques of machine learning.

Overall, ML usually includes three different groups of algorithms: supervised learning (SL), unsupervised learning (UL), and reinforcement learning (RL) [10] (Figure 1). In the SL, an algorithm is trained on the associations between the input variables and the outputs (of a training set) known before the training phase and represented by a group of labelled information. Upon such association, SL attempts to associate “new” inputs (i.e., the ones from a test set) to unknown outputs. In the UL, the information are unlabelled (i.e., the output is not known) and grouped based on dissimilarities and correspondences. Both these techniques are retrospectively used to analyse medical data and are employed for diagnosis, prognosis, genomic evaluation, relapse checking, and chemotherapy response assessment [11]. Finally, RL is adapted to different activities with respect to various environmental conditions in order to figure out the scenario represented by a non-static clinical situation. As an example, this might be employed for therapeutic protocols, where dosages are selected along with the clinical response and the side effects of treatments to generate a therapeutical approach that suits the specific patient [12]. Multivariate data analysis is also a possible approach of ML, where data involving more than a single type of measurement are present or where more than one dependent variable is analysed together with other variables.

On the other hand, as mentioned above, DL is a subgroup of ML that is inspired by the neurological structure and, in some instances, the functioning of the brain. With respect to other ML techniques, the neural network structure the DL is based on supports the possibility to exponentially scale up with the rising amount of data typical of the “big data” era [13], making DL exceptionally convenient for solving complicated computational problems, such as large-scale image analysis [13], or to obtain non-linear reports from a large quantity of data [14]. According to the brain structure, a deep neural system is composed by millions of computing neurons ordered into successive layers. Inside each layer, a neuron is related to other neurons in the layer before it, from which it collects information, and neurons in the layer after it, to which it delivers information. As such, a neural network improves each sample before transporting the data down to all subsequent layers. These data are then modified millions of times before they arrive at the last layer, which makes up the final result.

Overall, there are different DL-based techniques, including Multi-layer perceptrons (MLP), recurrent neural networks (RNN), and convolutional neural networks (CNN). MLPs represent a simple kind of neural network where neurons are structured in sequential layers so that information move via the system in one direction, using backpropagation of the information for learning purposes. However, MLPs are exposed to overfitting [15]. RNNs analyse an input sequence one item at a time while storing memory of all previous components. This technique is generally employed for studying sequential information, including DNA sequences. Finally, CNNs are able to learn invariant features and to represent spatial correlations from image data. They include ranked hierarchies, where the circulation of inputs varies throughout the course of learning [16].

**Figure 2:**
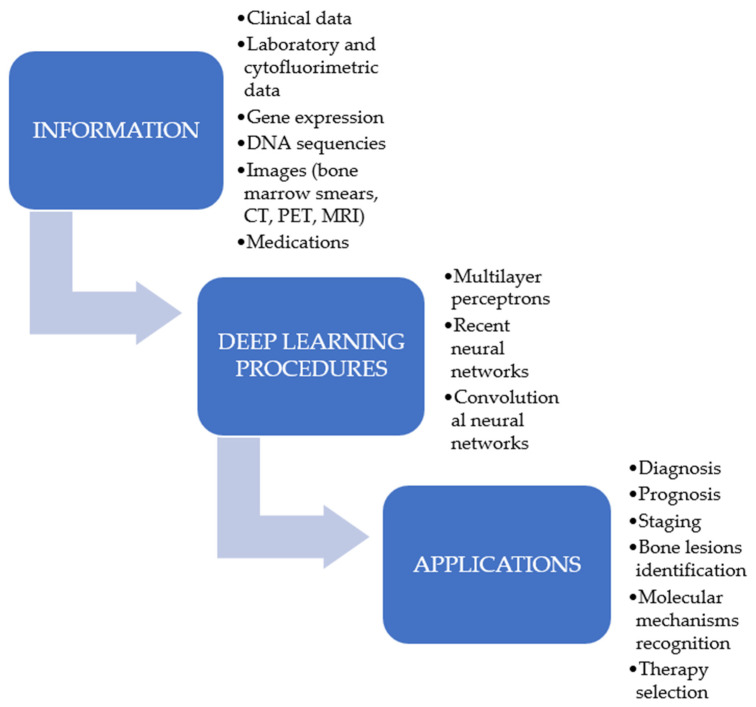
Possible applications of deep learning procedures in multiple myeloma research.

In the last few years, the application of ML algorithms and DL procedures to detect tumours has been progressively increased [17,18,19,20] (Figure 2), as they have the capability to use a mixture of proteomic, genomic, histopathology data, or images to manage cancer patients. However, the aim of DL or ML is not to replace the human ability but to provide a decision support to oncologists in their practice [21]. These procedures have been demonstrated to represent an advantageous support not only in the area of solid neoplasms but also in the management of the haematological patients. Recently, several reports have shown their importance in the diagnosis, prognosis, and therapeutic evaluation of haematological neoplasms. In light of such considerations, the purpose of this review is to present the most recent results concerning the use of these computational techniques in the management of a particular form of haematological neoplasia, namely the multiple myeloma (MM). MM was selected since it represents a haematological tumour provoked by the clonal proliferation of plasma cells with an increasing prevalence, accounting for more than 140,000 subjects diagnosed per year. Furthermore, whereas the survival after a diagnosis of MM has enhanced, the tumour has basically a negative outcome [22,23].

## 2. Machine Learning and Multiple Myeloma Diagnosis

Computerized tumour diagnosis is one of the most relevant fields of medical AI uses. Traditional ML techniques, such as k-Nearest Neighbour (kNN), Random Forests (RFs), Support Vector Machine (SVM), Gradient Boosting decision trees (GBDT), NB, and Artificial Neural Networks (ANNs) were employed for the computational diagnosis of MM (Table 1).

MM is a malignancy of plasma cells with composite pathogenesis, and its diagnosis and staging needs the combined analysis of serology, genetic, morphology, immunophenotypic, histology, and radiology information. The deferment in the diagnosis could delay the therapy, provoking the onset of severe problems [34].

Increased tumour load and the onset of organ damages cause a decreased survival [35]; for this reason, there is an increasing consideration about the use of AI methods for the diagnosis of MM.

According to the literature and considering the ability to manage problems of diagnosis and categorization, the GBDT is considered as a valid ensemble learning system [36]. In a study, aiming to increase the diagnostic percentage of MM, a ML model was implemented. A total of 4187 blood and biochemical exams were collected, which include 1741 results of MM patients and 2446 data from non-myeloma individuals, such as patients affected by renal or hepatic diseases, infectious diseases, or rheumatic diseases. The data collected were split into training and test subsets with the ratio of 4:1 while linking creatinine, calcium, haemoglobin, albumin, immunoglobulin, total protein, and the proportion of albumin to globulin data. An assistant diagnostic model of MM was created by GBDT, SVM, Deep Neural Networks (DNN), and RF. The methods were compared by means of the receiver operating characteristic (ROC) curve. All the ML algorithms evaluated (RF, DNN, and GBDT) worked fine. However, GBDT had the highest accuracy (92.9%), replicability (90.0%), and F1 score (0.915) for the MM patients. The maximized area under the ROC was calculated, and the results of GBDT (AUC: 0.975; 95% confidence interval (CI): 0.963–0.986) outperformed those of SVM, DNN, and RF. Therefore, the approach based on ML fed by standard laboratory findings can correctly identify MM, enhancing the percentage of an early detection [24].

Moreover, computational systems seem to be able to completely modify the reliability of the typical diagnostic techniques of MM, such as the morphological analysis of the bone marrow and its evaluation through an immunophenotypic study.

Bone marrow aspirate (BMA), with its differential cell counts (DCCs), is essential for the categorisation of hematologic conditions. In fact, disease classifying benchmarks are established on different rates of plasma cells for monoclonal gammopathy of undetermined significance (MGUS), including smouldering myeloma and MM. Although manual evaluation is considered to be the gold standard, it is a time-consuming procedure, and it is exposed to errors. A trustworthy computerised counter has to be created although digital imaging combined with ML algorithms is an extremely encouraging technique to this end. A recent study developed a ML algorithm to identify BM cells [25]. Using a web-based technique, over 10,000 cells taken from slide images of BM were manually interpreted. Authors performed a two-phase identification and categorisation method allowing customization and increased categorisation accuracy. Their algorithms obtained high accuracy in discovery and categorisation employing non-neoplastic samples. Analysing small series of MM tests, it similarly displayed excellent identification, categorisation, and classification accuracy. Therefore, the authors concluded that this procedure had the ability to support in MM detection, possibly influencing the clinical practice [25].

Comparable findings have been found by using specific computerised methods to the immunophenotypic study. Cellular characteristics can be studied and counted on a single MM cell by flow cytometry, which has been demonstrated advantageous not only for identification but also for risk evaluation and treatment assessment [37]. Furthermore, it should be reminded that, with technical developments, the number of antigens that can be evaluated at the same time by cytofluorimetric analysis has increased. Various computerized techniques for immunophenotypic study have been implemented [26,38,39].

In a different study, authors used a computational approach to discriminate MGUS smouldering multiple myeloma (SMM) and MM. Specific antigens recognized via diverse cluster of differentiation, such as CD27 and CD38, were differently recognized in MGUS and MM. By employing a gradient boosting machine technique, they displayed that the rate of clonal PCs and the ratio of PC/CD117 positive precursors were the more relevant factors to discriminate MGUS and MM. Lastly, they generated an algorithm allowing a predictive categorisation ≥95% when PC alterations were supposed, without any misclassification between MGUS and SMM samples. Finally, they proved the efficacy of this algorithm in an independent group of PC alterations [27]. However, prospective analysis should be performed to increase the quality of standardization [40,41].

Finally, it was described the chance to diagnose MM employing serum-based laser-induced breakdown spectroscopy (LIBS) and ML techniques. LIBS is a simple and reliable spectroscopic method that evaluates the atomic or molecular fingerprint due to emanations of plasmas produced by directing a pulsed laser on the samples [42]. Recently, LIBS has been used for the analysis of various biological samples, including cancer cells [43,44].

Chen et al. suggested the possibility to diagnose MM employing LIBS combined with quadratic discriminant analysis (QDA) and kNN [28]. In a different work, the serum samples of MM subjects in different stages of disease and normal subjects were analysed with a Q-switched Nd:YAG laser [29]. The serum-LIBS spectra were compared between the MM subjects and controls and among different stages of MM disease. Different ML techniques, including ANN, kNN, and SVM, were employed. Cross-validation was utilised to estimate the capacity of the discrimination systems concerning accuracy, precision, and area under the curve (AUC) of ROC. All the classifiers tested obtained analogous results with an accuracy over 90% for both diagnosis and staging of MM samples. These data demonstrate that LIBS combined with ML techniques can be used as a cheap, rapid, and reliable method for the diagnosis of MM [29].

### 2.1. Machine Learning and Bone Lesions Identification in Multiple Myeloma Patients

The detection of lithic bone lesions is pivotal in the diagnosis, prognosis, and therapeutic choice in MM patients [45]. Imaging techniques are of extreme relevance to recognize bone lesions. Overall, it is mandatory to establish the stage of the disease and to evaluate the therapy response. As such, radiographic skeletal investigation is commonly used in the study of MBD, whereas other techniques include computed tomography (CT), magnetic resonance imaging (MRI), 18F-FDG PET and, more recently, the employ of a targeted PET tracer, the 68Ga-Pentixafor [46,47,48,49,50].

However, even with the most sophisticated techniques, MM bone alterations remain challenging, and the identification of small lesions on hybrid imaging is susceptible to error.

Focusing on the existing literature, a study used DL techniques to computationally merge different systems, such as PET and CT, for MBD identification in a 3D mode. Two CNNs systems, V-Net and W-Net, were employed to segment and identify the MBD. The feasibility of DL for MBD identification by 68Ga-Pentixafor PET/CT was first established on digital phantoms produced employing realistic PET simulation procedures and afterwards by a real 68Ga-Pentixafor PET/CT examination of MM subjects. The findings demonstrated that DL procedures can provide multimodal data, and the W-Net achieved the best outcome for segmentation and lesion identification. It was also reported that W-Net outperformed traditional ML systems, including SVM, RF, and k-NN [30] (Table 1).

In a different study, Mesguich et al. aimed to create a system based on PET and CT images that could improve the identification of MBD via 18-FDG PET/CT [31]. Visual evaluation of PET/CT was performed by two different nuclear medicine physicians. Spine volumes on CT and PET were segmented, and a selection of the best features was performed using RF features relevance combined with correlation analysis. ML algorithms were then trained on the selected features with cross-validation, and the final model was tested on an independent test set. Eighteen patients presented relevant disease according to MRI. The accuracy and specificity of visual analysis were 75% and 70%, respectively, with a sufficient kappa coefficient of consensus of 0.6. However, the agreement between readers was modest and there was some intra-observer discrepancy at two different readings. Moreover, accuracy was only slightly enhanced after consensual reading.

On the training set, RF classifier achieved the greatest mean accuracy of 0.91 with an AUC of 0.90 for advanced MBD diagnosis. On the independent test set, the system obtained an accuracy of 80% [31]. Interestingly, the five biomarkers employed for the analysis were extracted from both CT and PET, highlighting the relevance of both imaging modalities for the diagnosis of diffuse disease. The lack of correlation in these five parameters between themselves suggests that they probably represent different information. Thus, radiomics evaluation of 18-FDG PET/CT images with ML algorithms overwhelmed the limits of visual investigation, offering an extremely precise identification of diffuse MBD in MM subjects.

Finally, an effort was performed to establish whether ML based on traditional MRI features is capable of differentially diagnosing MBD and different cancer metastasis of the lumbar vertebra [32]. After regression analysis, employing the least absolute shrinkage and selection operator algorithm, several ML systems were employed, such as Naïve Bayes (NB), SVM, KNN, RF, and ANN, using 10-fold cross validation, and the results were analysed through a confusion matrix. To evaluate the accuracy and specificity of the systems, Matthews correlation coefficient (MCC) was also employed. Among the classifiers, the ANN classifier from the T2WI images obtained the best results (MCC = 0.605) in the validation cohort, with accuracy, sensitivity, and specificity of 0.815, 0.879, and 0.790, respectively. Furthermore, the authors tried to discriminate MM lesions and metastasis subtypes. However, although ML classifiers demonstrated an acceptable efficacy in discriminating MM bone lesions from those of cancer metastasis, their performance in separating MM from other metastasis subtypes was modest [32].

The advanced computational techniques have also been used in other experimental studies performed on MM patients with innovative techniques. The introduction of surface-enhanced laser desorption/ionization time-offlight mass spectrometry (SELDI-TOF-MS) has offered the tools to evaluate a wide range of proteins in patient samples [32,51,52]. This technique, in association with informatics data evaluation, is extremely advantageous in several medical conditions, such as in the study of the MBD in MM patients. SELDI-TOF-MS was employed to check markers suggestive of bone alterations in MM subjects. Serum samples from 48 MM subjects (half with more than three lithic lesions and half without bone alterations) were evaluated employing copper ion-loaded immobilized metal affinity SELDI chip arrays. The spectra achieved were normalized, and mass peaks with mass-to-charge ratios (*m*/*z*) between 2000 and 20,000 Da were classified. Results were evaluated employing partial least squares discriminant analysis (PLS-DA) and RF. The PLS-DA system had a prediction accuracy between 96–100%, whereas the RF system obtained a specificity of 87.5% [33].

### 2.2. Machine Learning and Multiple Myeloma Prognosis

Evaluation of the prognosis is one of the main topics within tumour investigation, where AI is supposed to offer a substantial advancement in the management of MM subjects [53] (Table 2).

The initial staging system for MM was suggested by Salmon and Durie in 1975 [62]. Subsequently, the International Staging System (ISS) and the Revised-ISS (RISS) were formulated [63,64]. However, the introduction of proteasome inhibitors (PSI) and immunomodulatory drugs [65] have modified the prognosis of these patients, and therefore, the risk stratifications based on Salmon and Durie system, ISS and RISS, must be revised. Applications of ML algorithms recognized different risk groups and allowed a greater estimation of the outcome. Due to the size and complexity of information, such investigations would be unachievable without the use of ML algorithms and DL architectures.

In a study, Farswan et al. employed ML techniques to elaborate a novel staging system, the Modified Risk Staging (MRS), for MM using few laboratory markers: haemoglobin, Beta2Microglobulin (β2M), albumin, calcium, and estimated glomerular filtration rate (eGFR) along with age [54]. The system was elaborated on a training data group of MM patients and ratified on two test data groups. A comparison between this system with ISS and RISS was carried out to evaluate its ability to predict progression free survival (PFS) and overall survival (OS). K-adaptive partitioning (KAP) was employed to discover novel significant levels for the parameters considered. Risk staging instructions, achieved by training a J48 classifier (a decision tree classification algorithm and one of the best ML algorithms to examine the data categorically and continuously), were employed to generate MRS. Novel levels were recognized for calcium, albumin, eGFR, β2M, and haemoglobin by employing KAP on their inhouse MM Indian (MMIn) cohort. On this dataset, MRS outperformed ISS for OS calculation but was equivalent in the prognosis of PFS [54].

A different attempt was performed by the Euroflow consortium, which employed a PCA-based system for minimal residual disease (MRD) evaluation in MM [55]. A group of 12 markers was evaluated in a series of diagnosis and follow-up MM BM samples, and the automated parameter separation (APS) system of the Infinicyt analysis software (PCA-based) was employed to calculate parameters that best differentiated between groups. The best six parameters (CD19, CD45, CD56, CD81, CD27, and CD117) were employed for MRD recognition, and APS was used to help recognition of MRD cell populations. This system, called the Next-Generation Flow (NGF), identified 47% of samples as MRD-positive. Overall, 25% of subjects recognized as MRD-negative by traditional eight-colour flow were MRD-positive by NGF. OS in NGF MRD+ subjects was considerably shorter than in NGF MRD patients. Although NGF did not represent a completely automatic classification of MRD cells, it demonstrated that such systems might increase the accuracy of expert evaluations [66].

Encouraging results have been also obtained by combining clinical and laboratory parameters with gene expression profiling (GEP). In a report, a ML system was used to analyse MM clinical and RNA-sequencing (RNAseq) information. A 50-variable RF system (IAC-50) was generated to calculate OS. The continuous rank probability score (CRPS) was evaluated as the integrated Brier score divided by time. This system included several variables: ISS stage, age, first-line therapy, and the expression of 46 genes. Remarkably, OS evaluation demonstrated that those patients cured with the best-expected drug combination survived longer than those subjects administered with other protocols. This was drastically relevant among MM subjects administered with a combined treatment, including bortezomib, an immunomodulatory drug, and dexamethasone [56].

The use of the ML algorithms could also allow the detection and validation of other prognostic parameters, including mRNA expression-based stemness index (mRNAsi). Stemness was described as the capacity for differentiation and proliferation from a pluripotent cell or a cancer cell. However, the processes triggering the proliferation and survival of MM stem cells are not clear. The stemness index for MM cells was evaluated by employing a new one-class logistic regression (OCLR) ML system, and it was recognized that mRNAsi was an independent predictor for MM [57], able to differentiate MM subjects into groups with different OS. A total of 127 stemness-correlated signatures employing weighted gene co-expression network analysis (WGCNA) were recognised. Pathway enrichment analysis revealed that these genes were essentially involved in the regulation of differentiation, cell cycle, and DNA repair. Employing the molecular complex detection (MCODE) algorithm, 34 critical signatures were found. Furthermore, the study performed an unsupervised clustering and ordered the MM patients into three different MM stemness (MMS) groups with different outcomes. Patients with samples in MMS group 3 had the highest stemness fractions and presented the worst outcome. In addition, the employ of the ESTIMATE algorithm to evaluate a different immune activity among the three MMS groups allowed to confirm a negative correlation between stemness and anti-MM immunity. Finally, they suggested a predictive nomogram that allows individualized evaluation of the three- and five-year OS possibilities [57].

### 2.3. Machine Learning and Prediction of Clinical Drug Response

In spite of the exceptional advancement in the therapy of MM thanks to the finding of new drugs [67,68,69], most subjects with MM relapse [67,68,69]. Large interindividual differences in the response to treatment represents the main shortcoming in obtaining persistent results in several tumours, including MM. This heterogeneity after therapy is due to the modifications in the expression of genes involved in chemoresistance [70,71,72]. Interpreting alterations in gene expression is consequently crucial to evaluate the future effectiveness of antimyeloma treatments and to avoid deferral in the right choice of different therapies. However, even though technical progresses in high-throughput drug screening in cells led to the production of a considerable volume of significant information, examination of such information remains troublesome.

Differential gene expression profile (GEP) analyses have offered GEP as an advantageous predictive marker of low- vs. high-risk MM. Nevertheless, until present, none of such profiles were drug specific [73,74,75].

For instance, proteasome inhibitors (PIs) are commonly used in the treatment of MM but can provoke relevant side effects, and response to therapy differs among patients [76]. Considerable attempts have been performed to find what characterises responders to PIs from non-responders, and several experimentations compared GEP of subjects reacting well or inadequately to the PIs administration. Recently, the genes identified were combined in a ML algorithm to predict the response to the treatment [73,74,75].

In a study employing a series of myeloma cell lines (MCLs) representative of the genetic heterogeneity of MM patients, an in-vitro chemosensitivity pattern in response to therapy with four PIs (bortezomib, carfilzomib, ixazomib, and oprozomib) as single agents was reproduced [58]. Using ML-based computational methods, including RF and Random survival forest, they recognized a 42-gene profile that could discriminate subjects based on their response (Table 2).

These findings suggest the use of in-vitro experimentation and ML-based techniques to determine prognostic markers of response and chemoresistance to PIs, but a key problem stems when a MM subject profits more from a PI than from a different therapy [77].

Ubels et al. projected a new system, Simulated Treatment learning signatures (STLsig), to extrapolate gene profiles that can predict therapy advantage for MM subjects at the diagnosis [78]. They employed STLsig to identify subjects for whom PIs therapy causes a better survival than different treatments.

In a group of 910 MM subjects, STLsig identified two gene complexes that can jointly predict good effects for the PI bortezomib [59]. In the group “benefit,” they found a Hazard Ratio (HR) of 0.47 in support of bortezomib, while among those in “no benefit,” the HR was 0.91. Furthermore, this profile also foresees efficacy for the PI carfilzomib, suggesting the non-exclusiveness to bortezomib. Several genes included in the profile are correlated to functioning systems of PIs or MM progression. Hence, STLsig can recognize gene profiles that might help in therapy selection for MM subjects and offer understanding into the mechanism behind therapy profit.

In a different experimentation, Borisov et al. described novel RNA sequencing profiles for 53 MM subjects marked with outcome on two different treatment protocols: bortezomib, doxorubicin, and dexamethasone (PAD) and bortezomib, cyclophosphamide, dexamethasone (VCD) [60]. Authors evaluated profiles for the good and bad responders and recognized five increases in the good responders: GRB14, MAF, FGFR3, IGHA2, and IGHV1-69. Then, authors used several ML algorithms to generate a classifier discriminating good and bad responders for two groups of patients: PAD + VCD and, distinctly, VCD. For each ML algorithm, the authors employed a data-trimming/pre-processing step using FloWPS system to increase effectiveness. Among the ML system, they used RF, linear SVM and ridge regression (RR), binomial NB, and MLP. Results demonstrated that the use of FloWPS dynamic data trimming was efficient for all ML algorithms employed in both groups and also in a prior MM bortezomib group of data. Nevertheless, the ML systems generated for the different groups of data did not allow cross-transferring, which is probably due to the different treatment protocols, investigational methodologies, and MM heterogeneity [60].

Finally, a Multi Learning Training approach (MuLT) joining supervised, unsupervised, and self-supervised learning systems was also proposed to evaluate the prognostic significance of heterogeneous therapy outcomes for MM. Venezian Povoa et al. reported that gene expression values increase the therapy sensitivity prognosis and wraps up genetic alterations discovered by Fluorescence in-situ hybridization analysis [61]. MuLT functioning was evaluated by cross-validation experiments, in which it displayed therapy sensitivity with 68.70% of AUC. Furthermore, the results suggested that, in around 17.07% of MM subjects, it could obtain better response to a different chemotherapy at the first line.

### 2.4. Machine Learning and Apoptosis in Multiple Myeloma

The high computational ability of the ML algorithms could also promote the explanation of some pathophysiological processes of the MM. Several experimentations have demonstrated that the intrinsic apoptotic system has an essential effectiveness in the MM therapies. The PI bortezomib has been reported to provoke programmed cell death in MM cells by producing an increase of proapoptotic BIM and a decrease of the pro-survival protein, MCL-1 [79,80], while MM cells quickly experience apoptosis upon genetic deletion of MCL-1 or BCL-2 [81] or use of BCL-2 or MCL-1 inhibitors [82,83,84]. Hence, in-depth knowledge of the programmed cell death dynamics in MM after therapy could influence the selection of better drug protocols able to destroy MM cells.

In a study, authors tried to recognize the best adjunct targets to destroy MM cells resistant to traditional treatments employing deep profiling by mass cytometry (CyTOF) [85]. They evaluated 26 controllers of cell signalling, mitosis, cell death, and MM-correlated pathways after therapy with dexamethasone or bortezomib. Time-resolved visualization algorithms and ML RF models defined predicted cell death paths and a ranking of factors that identified MM cell survival or programmed cell death after therapy. Among these factors, increased amounts of MCL-1 and phosphorylated cAMP response element-binding protein were identifying attributes of cells persisting after drug administration. Thus, ML algorithms might be useful to detect clinically effective drug combinations and to evaluate the patient response to treatment.

## 3. Conclusions, Challenges, and Perspectives

Latest progresses in producing a constantly growing amount of biomedical information and the increasing computational ability to analyse such data suggest the possibility of a quicker fusion of data technology and the clinical universe. At the connection of composite biomedical information and computational evaluation, ML and DL assume a relevant role due to improvements in hardware elements and software knowledge [86,87].

Together with the improvement obtained in the study of MM via the use of ML and DL, novel study possibilities are opening. For instance, an exciting new approach for studying the MM BM milieu is represented by spatial transcriptomics, which allows to quantize gene expression in single cells or regions while preserving their positional representation and so maintaining spatial heterogeneity of gene expression [88,89]. Considering the complexity of this information, DL methodologies are appropriate for this evaluation and analysis. For instance, by combining histopathology descriptions and spatial transcriptomics, DL might calculate local gene expression from a simple smear, as proven by ST-Net, a neural network able of foreseeing gene expressions in breast cancer using tissue slides [90].

However, there are several limitations to the extensive application of ML and DL procedures in clinical practice. First of all, data mutability is a main problem for using such systems in haematology. In fact, it is presently uncertain how these procedures would operate with inter- and intra-laboratory variability. In addition, there is a great variety of data normalization (FPKM, TMM, TPM) and data conversion approaches (log, linear, etc.) with applications in several programming languages, causing a huge amount of processing pathways that could provide the same findings but without any recognised process to guarantee the accuracy of a methodology.

Moreover, ML algorithms could retrieve tricky correlations from large groups of data, but there is a serious deficiency of understanding about their causal connections [91]. Furthermore, for DL to be employed in clinical practice, the systems need to be built up purposely to improve the clinical workflow.

However, other pivotal issues have to be declared, including the unambiguousness of the systems, which correlates to the data utilised, while the reliability, functioning, and weaknesses of a single model have to be assessed. Further on, other factors should be evaluated, including ethical and medico-legal issues and a still largely unmet need of full validation and integration of AI systems in the existing framework for clinical decision management. For instance, a key question might be if AI is able to replace physicians in the observation, characterization, and quantification tasks that they presently carry out employing their cognitive skills. For this question, the answer is likely to be a negative one, as it is essential to highlight that the final decision concerning patient diagnosis is still independent, and the responsibility remains with the physicians, not AI systems. In fact, one of the main biases that can hinder the employment of AI in clinical activity is the automation bias, which can be described as the tendency to support a machine-generated diagnosis over the evidence derived from scientific knowledge and the physician’s own expertise [92].

In their activities, physicians should realize how to better employ and interpret AI algorithms, including in which situations a medical AI should be used and how much confidence should be assigned in an algorithmic conclusion. Although AI creates new prospects, the basic principles of the clinical reality do not change. In order to effect real impact on patient care, AI-based research in medical activity must observe the first principles in medical science. Research postulates, both AI-based or not, must be clinically appropriate and accountable in the clinical domain [93]. This is a challenge, and evaluation tools are still very much under development, making it necessary for medical AI to be trained in appropriate environments, using optimized techniques, and on complete datasets [94,95].

In addition, the process of AI systems integrated into big data networks produces legal and ethical problems correlated to patient-specific consent, privacy protection, data sharing, and the accessibility of multi-layered access to fully or partially anonymized health information [96].

Finally, present AI techniques are not transparent in their elaboration processes; that is, their interlocutors might have no well-defined representations of how AIs have reached a given conclusion: this could produce “trust issues,” especially when critical choices should be taken based on these conclusions. Moreover, upcoming studies on the ethical implementation of AI in medical evaluation should consider patients’ perception of these instruments and estimate under which conditions patients may feel “put aside” by their doctor because health recommendation and therapy are provided by autonomous technologies [97]. In conclusion, large amounts of data on MM have been now collected and are quickly growing. The use of ML and DL procedures may further increase our knowledge, help to obtain a better understanding of the mechanisms of myelomagenesis, and allow a better management of the MM patients. In the near future, AI is expected to participate more and more in decision-making processes, as desirable features of AI systems include the ability to perform simple, yet repetitive and time-consuming tasks and optimization of workflow management, leaving more time for clinical patient supervision [98].

## Figures and Tables

**Table 1:** Artificial intelligence (AI) applications in multiple myeloma diagnosis, and bone lesions identification.

Diagnosis			
Parameters	AI Tools	Ref.	Key Findings
Blood and biochemical exams	Gradient boosting decisional tree	[24]	A ML approach on standard laboratory findings enhances the percentage of early detection
Differential cell counts of bone marrow aspirate	VGG16 convolutional network	[25]	Bone marrow aspirate differential counts employing ML techniques
Cytofluorimetric analysis of bone marrow aspirate	FlowCAP	[26]	Computerized methods for cytofluorimetric analysis
	Gradient boosting machine technique	[27]	Classification of plasma cell dyscrasias by combining AI and flow cytometry
Laser-induced breakdown spectroscopy analysis	Quadratic discriminant analysis, k-Nearest Neighbour	[28]	Diagnosis of malignancies using serum-based laser induced breakdown spectroscopy and chemometric methods
	K-Nearest Neighbour, Support Vector Machine, Artificial Neural Networks	[29]	Diagnosis of malignancies using serum-based laser induced breakdown spectroscopy in combination with ML methods can serve as fast technique for MM diagnosis and staging
**Bone Lesions Identification**			
**Techniques**	**AI tools**	**Ref.**	**Key findings**
PET and CT	Convolutional neural network (v-Net, w-Net)	[30]	^68^Ga-Pentixaflor PET/CT and DL techniques to detect MM whole-body bone lesions
PET and CT	Random Forest	[31]	Radiomics analysis of 18-FDG PET/CT image with ML overcame the limitations of visual analysis
MRI	Naïve Bayes, Support Vector Machine, k-Nearest Neighbour, Random Forest, Artificial Neural Networks	[32]	ML radiomics is able to differentiate between MM and metastasis subtypes of lumbar vertebra lesions
SELDI-TOF-MS (mass peaks with mass-to-charge ratios)	Random Forest, Partial least squares discriminant analysis	[33]	SELDI-TOF-MS and ML tools discriminate MM patients with and without skeletal involvement

SELDI-TOF-MS, Surface enhanced laser desorption/ionization time-offlight mass spectrometry.

**Table 2:** Artificial intelligence (AI) applications in multiple myeloma prognosis and prediction of response to treatment.

Prognosis				
Parameters	AI Tools		Ref.	Key Findings
Laboratory parameters	k-adaptive partitioning		[54]	AI-supported modified risk staging for multiple myeloma
Beta2microglobulin	Infinicyt software		[55]	Next-Generation Flow and ML for highly sensitive detection of minimal residual disease
Gene expression profile, ISS stage, first line therapy	Random Forest		[56]	Survival prediction and treatment optimization using ML models based on clinical and gene expression data
mRNA expression-based steamness index	One-class logistic regression		[57]	Analysis of gene expression via one-class logistic regression ML identifies stemness features in MM
**Prediction of Response to Treatment**				
**Drugs**	**Parameters**	**AI tools**	**Ref.**	
Bortezomib, carfilzomib, ixazomib, oprozomib	Gene expression profile	Random Forest	[58]	A gene expression signature distinguishes resistance to proteasome inhibitors
Proteasome inhibitors	Gene complex	Simulated Treatment learning signature	[59]	Gene networks constructed using simulated treatment learning can predict proteasome inhibitor benefit
PAD, VCD	Gene evaluation	Random Forest, Support Vector Machine, Ridge Regression, Binomial Naïve Bayes, Multi-layer perception	[60]	ML applicability for classification of chemotherapy response using 53 MM RNA-sequencing profiles
Five first-line treatments (Bor-Cyc-Dex, Bor-Dex, Bor-Len-Dex, Len-Dex, Non-treatment)	Clinical markers, gene evaluation	Multi Learning Training approach	[61]	ML predicts treatment sensitivity in MM based on molecular and clinical information coupled with drug response

Bor, bortezomib; Cyc, cyclophosphamide; Dex, dexamethasone; Len, lenalidomide.
